# *Perrottetiawichmaniorum* (Dipentodontaceae), a new species from Kauaʻi, Hawaiian Islands

**DOI:** 10.3897/phytokeys.115.30657

**Published:** 2019-01-30

**Authors:** David H. Lorence, Warren L. Wagner

**Affiliations:** 1 National Tropical Botanical Garden, 3530 Papalina Road, Kalāheo, HI 96741, USA National Tropical Botanical Garden Kalāheo United States of America; 2 Department of Botany, Smithsonian Institution, PO Box 37012, Washington, DC 20013-7012, USA Department of Botany, Smithsonian Institution Washington United States of America

**Keywords:** Dipentodontaceae, *
Perrottetia
*, Hawaiian Islands, Kaua‘i, conservation, IUCN Red List, endangered

## Abstract

*Perrottetiawichmaniorum* Lorence & W. L. Wagner, **sp. nov**. is described as endemic to Kaua‘i. It differs from its Hawaiian congener *P.sandwicensis* by its larger, thicker, more densely hirtellous-villosulous rugose leaves with a smaller length:width ratio [1.5–1.7:1], larger inflorescences with usually four degrees of branching with moderately to densely hirtellous axes, and flowers with glabrous petals. This new species falls into the Endangered (EN) category when evaluated using the IUCN Red List criteria for endangerment based on its small area of occupancy, a decline in the extent and quality of its habitat, and number of mature individuals.

## Introduction

*Perrottetia* Kunth is a genus of 16–17 species of shrubs or small trees distributed from China, Malesia, Australia, and the Pacific to Central and South America, with a center of diversity in Colombia (Ding 1962; Lundell 1985; Mabberley 2017; Sánchez Montano and Fernández Alonso 2000). Formerly *Perrottetia* was often included in Celastraceae (e.g., Ding 1962; Matthews and Endress 2005). However, recent molecular evidence places it in Dipentodontaceae in the small order Huerteales (Zhang and Simmons 2006; Worberg et al. 2009). In their treatment of *Perrottetia* in the Hawaiian Islands, Wagner et al. (1990, 1999) recognized a single species, *P.sandwicensis* A. Gray, occurring on all the main islands except Ni‘ihau and Kaho‘olawe. Subsequent field studies and examination of herbarium specimens from Kaua‘i revealed the presence of a second previously undescribed species. We here describe *Perrottetiawichmaniorum* Lorence & W. L. Wagner, which differs from *P.sandwicensis* by its larger, thicker, more pubescent and rugose leaves with a smaller length:width ratio [1.5–1.7:1], larger inflorescences with up to four degrees of branching and moderately to densely hirtellous axes, and flowers with glabrous petals. This new species is known only from wet forests in the Hanalei District of northern Kaua‘i, where it sometimes grows sympatrically with *P.sandwicensis* and apparently occasionally hybridizes with it. We found the characters separating the two species to be well differentiated in all populations, in addition to their growing sympatrically in some areas. However in both species, smaller plants or branches with smaller leaves may have correspondingly smaller inflorescences that are less highly branched.

The affinities of *Perrottetia* species in the Hawaiian Islands are obscure and would benefit from molecular studies to resolve phylogenetic relationships. Also, the breeding systems of both species should be studied in more detail in the field. The breeding system of *P.sandwicensis* was first described as being polygamodioecious by Gray (1854). That of *P.wichmaniorum* as being dioecious is based on our own observations. The breeding system for both species is characterized based on morphology and not experimental results that should be considered in future work.

### Key to the Hawaiian Species of *Perrottetia*

**Table:** 

1	Leaves with blade broadly elliptic, broadly ovate, or suborbicular, 7–18.5 cm long, 4.5–10.5 cm wide [length:width 1.5–1.7:1], stiffly chartaceous to subcoriaceous, rugose with venation depressed adaxially and prominulous abaxially; inflorescences with usually four degrees of branching, the axes moderately to densely hirtellous; petals glabrous	**1. *Perrottetiawichmaniorum***
–	Leaves with blade ovate, elliptic, or oblong-elliptic, rarely obovate or broadly elliptic (Maui), 7–19 cm long, 2.5–9.5 cm wide [length:width 1.8–2.7:1], chartaceous, smooth, the venation not depressed adaxially nor prominulous abaxially; inflorescences with up to three (rarely to four on Kaua‘i) degrees of branching, the axes glabrate to densely brown villosulous; petals with ciliolate margins	**2. *Perrottetiasandwicensis***

#### 1. Perrottetia wichmaniorum

Taxon classificationPlantaeStylommatophoraStreptaxidae

Lorence & W. L. Wagner sp. nov.

urn:lsid:ipni.org:names:77194235-1

Figs 1 , 2A–C

##### Diagnosis.

*Perrottetiawichmaniorum* is similar to *P.sandwicensis* from which it differs by its larger, thicker, more pubescent and rugose leaves with a smaller length:width ratio [1.5–1.7:1], larger inflorescences with usually four degrees of branching with moderately to densely hirtellous axes, the staminate inflorescences 12.5–14 cm long, 10–13 cm wide, the pistillate inflorescences 9–13 cm long, 6–13 cm wide, and flowers of both sexes with glabrous petals.

##### Type.

**Hawaiian Islands: Kaua‘i**: Hanalei District, Nā Pali-Kona Forest Reserve; upper Hanakoa Valley north of Pihea Peak, 3900–4000 ft, 21 Dec. 1988 (pistillate), *T. Flynn & D. H. Lorence 3246* (Holotype: PTBG-014949!; Isotypes AD!, F!, MO!, MU!, SING!, US!).

##### Description.

Sprawling dioecious small tree 1.5–3 m tall with a dense crown or sometimes a shrub; branches when fresh green or sometimes purple or maroon-tinged, densely villous-hirtellous with pale brown hairs 0.2–0.5 mm long. Leaves spirally arranged, dark green except petioles and veins usually purple or maroon-tinged when fresh; blade thickly chartaceous to subcoriaceous, broadly elliptic, broadly ovate, or suborbicular, 7–18.5 cm long, 4.5–10.5 cm wide [length:width 1.5–1.7:1], adaxially glabrous, rugose with secondary, tertiary, and quaternary venation depressed, abaxially with venation prominulous, densely hirtellous-villosulous along costa and on veins, the hairs rufous, 0.2–0.6 mm long, the secondary veins 9–12 on each side, the axils occasionally with hair tufts, the tertiary veins conspicuously anastomosing, base broadly cuneate to obtuse or truncate, apex acute to rounded, the tip short acuminate, margin serrate-dentate, the teeth 0.5–1 mm long, tips indurated; petiole 1.5–5 cm long, brown villosulous or glabrate; stipules linear-oblong, 2–2.4 mm long, glabrous, deciduous. *Inflorescences* of both sexes axillary or occasionally infrafoliar, paniculate cymes with usually four degrees of branching, pyramidal, purple or maroon-tinged, the staminate 12.5–14 cm long, 10–13 cm wide, the pistillate 9–13 cm long, 6–13 cm wide, both with the peduncle 2–4 cm; axes densely rufous hirtellous, the hairs 0.2–0.3 mm long, bracts glabrous, ovate-elliptic or subulate, those on primary branches 1.8–2.0 mm long, those on secondary branches 1.2–1.5 mm long, those on tertiary branches 1–1.2 mm long, pedicels with 1–3 glabrous linear-oblong to subulate bracts 0.5–1.5 mm long; flowers on glabrous pedicels 0.5–1 mm long, hirtellous below articulation with ultimate axis. Staminate flowers with sepals and petals 5, spreading at anthesis, sepals purplish green, ovate, 1.0–1.2 mm long, apex acute to acuminate, glabrous, margins entire, petals white, ovate, similar to sepals in size but thinner, glabrous, margins entire; stamens on filaments 2–2.5 mm long, anthers transverse-ellipsoid, 0.5–0.6 mm long, 0.6–0.7 mm wide; pistillode 0.8–1.0 mm. Pistillate flowers with sepals and petals 5, glabrous, subequal, not spreading at anthesis, sepals reddish purple, ovate-deltate, 1–1.4 mm long, apex acute, petals white, ovate, similar in size to sepals but thinner; pistil conical, 1.2–1.5 mm long, stigma lobes 2(3), papillose; staminodes 5, 0.3–0.4 mm long. *Infructescence* 8–14.5 cm long, 6–14 cm wide. *Fruit* a globose berry 2.5–4 mm in diameter, ripening red or purple tinged, glabrous. *Seeds* (2‒)4, subglobose, tan, 1.2–1.5 mm long, with thin aril, the testa rugose-reticulate.

**Figure 1.**
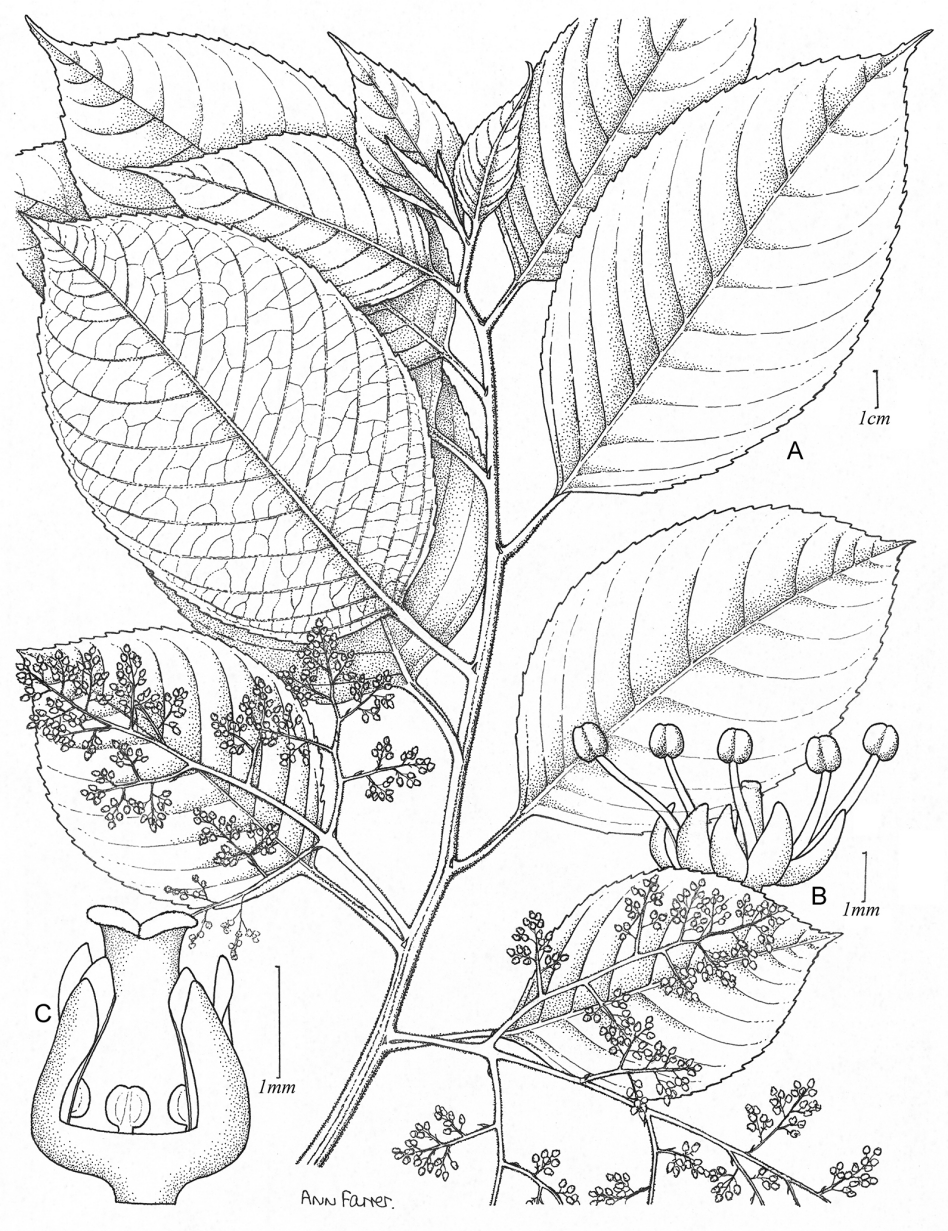
*Perrottetiawichmaniorum* Lorence & W. L. Wagner **A** Habit, branch with pistillate inflorescences **B** Staminate flower **C** Pistillate flower. **A, C** drawn from holotype *T. Flynn & D. H. Lorence 3246* (PTBG), **B** from *T. Flynn & L. Hume 3292* (PTBG).

**Figure 2.**
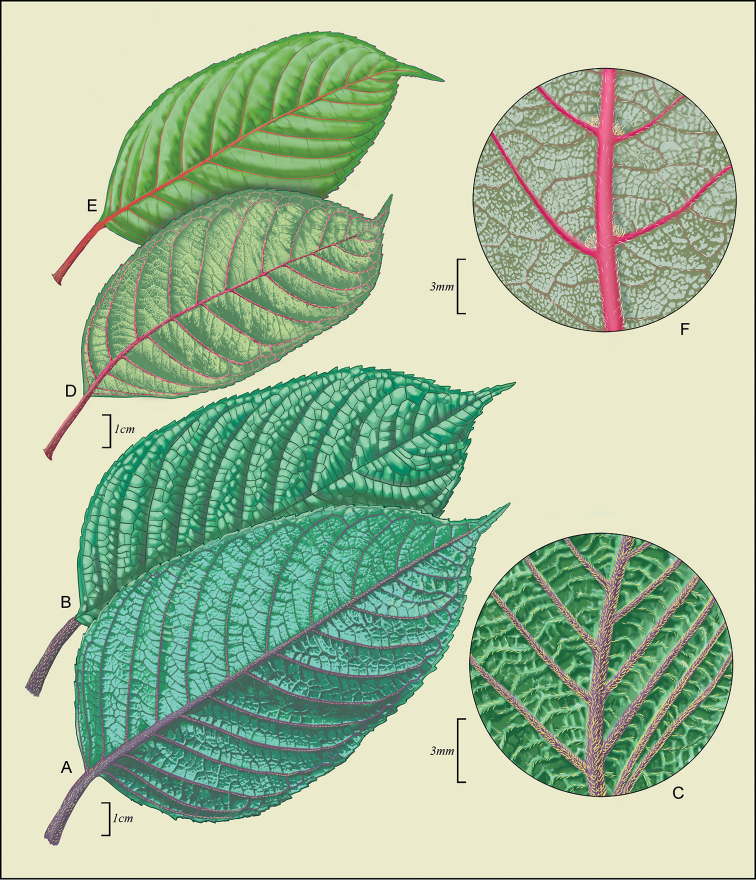
*Perrottetiawichmaniorum* (**A–C**) **A** Abaxial leaf surface **B** Adaxial leaf surface **C** inset of abaxial leaf surface. Drawn from isotype and field images of *T. Flynn & D. H. Lorence 3246* (US), and *W. L. Wagner et al. 6265* (US). *Perrottetiasandwicensis* A. Gray (**D–F**) **D** Abaxial leaf surface **E** Adaxial leaf surface **F** inset of abaxial leaf surface. *Drawn from D. Herbst 871* (US). Field images from Forest and Kim Starr [http://www.starrenvironmental.com/resources/] were used to augment illustration, especially colors: Ko‘olau Gap, Maui [#110713-7251 (25006081321_ac9bdd7fdb_o), #110713-7252 (24731716429_d7c44c451a_o), and #110713-7253 (25006085211_f687c67af8_o)]; Road to Lower Kula Pipeline Haiku Uka, Maui [#170520-0692 (34385766464_bb0d9bc7d0_o), #170520-0693 (34385767704_f28a1b34d7_o)].

##### Distribution.

**Figure 3.**
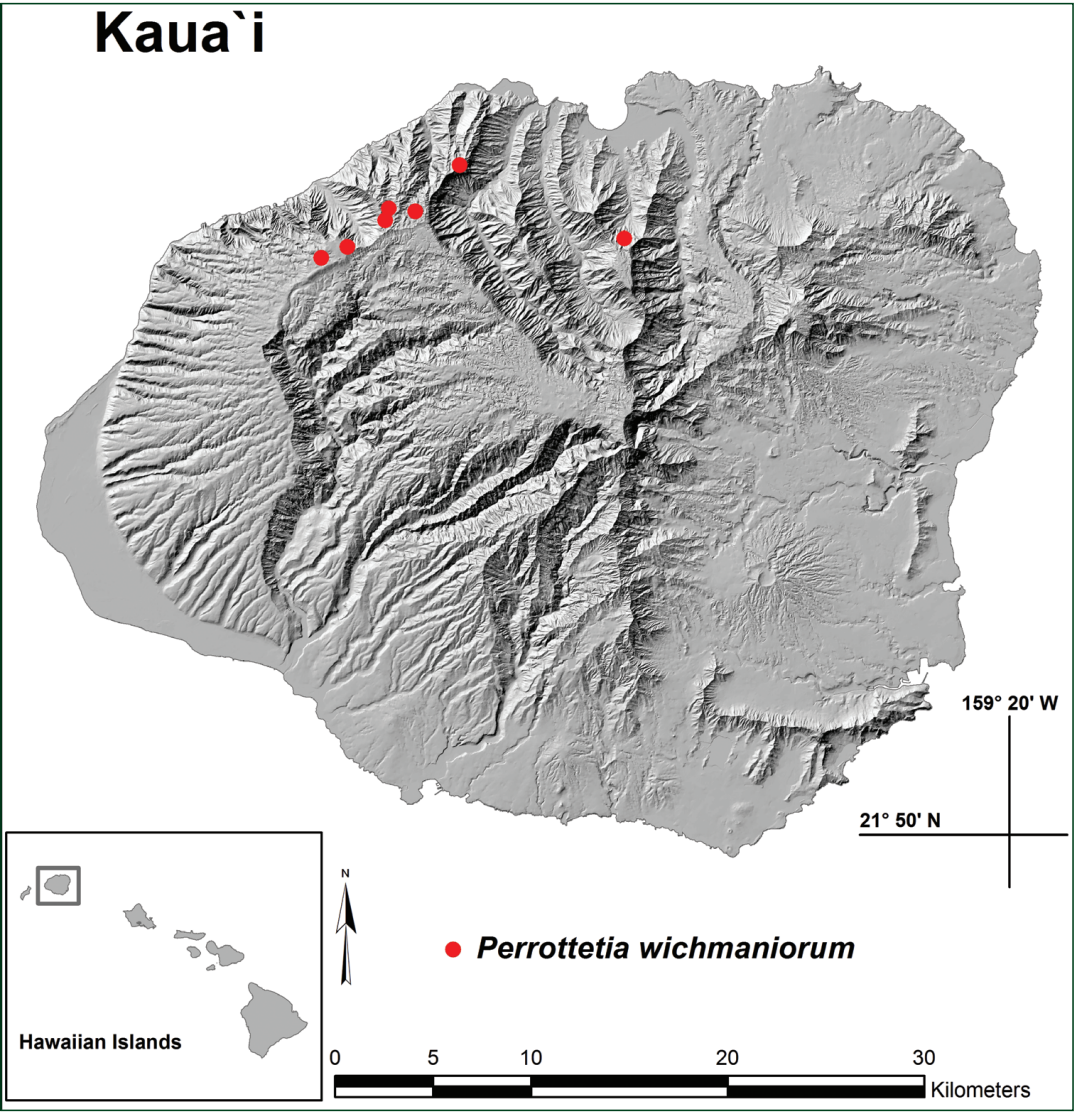
Distribution map showing known locations of *Perrottetiawichmaniorum* on Kaua‘i.

Hawaiian Islands, northern Kaua‘i, known only from the Hanalei District, growing along streams or on windward upper valley slopes and summit areas above the valleys at c. 740–1280 m (Fig. 3). Collections are known from the upper Waioli Valley in the east and along the Nā Pali Coast to Awa‘awapuhi Valley in the west, although they likely also occur in intervening areas with suitable habitat.

##### Habitat and ecology.

This new species grows in diverse wet forest with species of *Metrosideros* Banks ex Gaertn. dominant, associated with species of *Cheirodendron* Nutt. ex Seemann, *Syzygium* Gaertn., *Psychotria* L., *Melicope* J. R. Forst. & G. Forst., *Broussaisia* Gaud., *Dubautia* Gaud., *Smilax* Tourn. ex L., *Astelia* Banks & Sol. ex R. Br., and diverse ferns including *Dicranopterislinearis* (Burm. f.) Underw., *Cibotium* Kaulf., *Sadleria* Kaulf., and *Diplazium* Sw. Flowering was observed from November to June and fruiting from January to August.

##### Threats.

Threats to this species include invasive alien plant species, primarily *Axonopusfissifolius* (Raddi) Kuhlm., *Buddlejaasiatica* Lour., Clidemiahirta(L.)D. Donvar.hirta, *Erigeronkarvinskianus* DC., *Hedychiumgardnerianum* Sheppard ex Ker Gawl., *Juncusplanifolius* R. Br., *Rubusargutus* Link, and *R.rosifolius* Sm. Habitat modification by introduced pigs (*Susscrofa*), blacktail deer (*Odocoileushemionus*), and goats (*Caprahircus*) also negatively impact this species. Although recorded as first naturalized on Kaua‘i as recently as 2004, *Buddlejaasiatica* is rapidly becoming a very serious invasive species in the same wet drainages and riparian zones in which *Perrottetiawichmaniorum* occurs.

##### Conservation status.

When evaluated using the World Conservation Union‘s IUCN Red List criteria for endangerment (IUCN 2012), *Perrottetiawichmaniorum* falls into the Endangered (EN) category, a designation recommended for taxa facing a very high risk of extinction in the wild. The species merits this designation by having a very small Extent of Occurrence (EOO) of ca. 35 km^2^, along with a small Area of Occupancy (AOO) of ca. 10 km^2^, and an estimated population of ca. 7200 individuals (K. R. Wood, pers. comm.). The formal IUCN coding system for our evaluation is EN, B1ab(i,ii,iii,v)+2ab(i,ii,iii,v), which indicates that *P.wichmaniorum* is subject to an inferred decline in its area of occupancy, in addition to a decline in the extent and quality of its habitat and number of mature individuals.

##### Etymology.

We take pleasure in naming this new species for Charles R. “Chipper” Wichman, Jr., who has served as Director and CEO of the National Tropical Botanical Garden (NTBG) since 2005, and his wife Hau‘oli Wichman, who has served alongside Chipper throughout his directorship, for both their service on behalf of the Garden and especially for their efforts to conserve the Hawaiian Flora. Chipper has also served as Director of NTBG‘s Kahanu Garden on Maui and Limahuli Garden on Kaua‘i, where the new species occurs in the Upper Limahuli Preserve.

##### Specimens examined (paratypes).

**Hawaiian Islands.** Kaua‘i: Hanalei District. Nā Pali-Kona Forest Reserve, upper Hanakoa Valley north of Pihea peak, *T. Flynn et al. 2937* (AD, F, PTBG), *T. Flynn & D. H. Lorence 3247* (BISH, PTBG, US); Kōke‘e State Park, Hwy 550 at mile 19 [southern rim of Kalalau Valley], along north side of road, *T. Flynn et al. 3257* (PTBG); Kōke‘e State Park, Awa‘awapuhi Valley west and below Hwy 550, *T. Flynn & D. H. Lorence 3260* (PTBG); Kōke‘e State Park, along Hwy 550 near mile 19 on edge of Kalalau Valley, *T. Flynn & L. Hume 3292* (PTBG); upper Hanakoa Valley just E of Kalalau Valley rim, *D. H. Lorence et al. 6312* (BISH, PTBG, US); upper Waioli Stream Valley, main waterfall, hanging valley between upper and lower falls on N face of Nāmolokama Mt., *D. H. Lorence et al. 7295* (PTBG); Kalalau rim, north, below Pu‘u o Kila, *K. R. Wood 1047* (PTBG); Kalalau rim, NW facing aspect, below Pihea Peak, *K. R. Wood 1132* (BISH, PTBG, US); upper Hanakoa valley from Pihea peak, southwest facing cliffs, *K. R. Wood et al. 2218* (PTBG); upper Hanakāpī‘ai drainage and the North Bog area along Wainiha Rim (ridge to Hono o nā pali), below in Hanakāpī‘ai head-water, east drainage, *K. R. Wood 5272* (PTBG); upper Hanakoa drainage north of Pihea peak, west of Moa‘alele, *K. R. Wood & S. Perlman 7453-A* (PTBG, US); Limahuli, upper south-east corner below Pali Ele‘ele, *K. R.Wood 7508* (PTBG); below Pihea Peak in Upper Hanakoa Valley, *W. L. Wagner et al. 6265* (PTBG, US).

##### Discussion.

This new species sometimes grows sympatrically with *P.sandwicensis* with which it occasionally hybridizes. Two examples of putative hybrids were recognizable by their intermediate leaf morphology and inflorescence structure, if fertile.

##### Putative hybrids.

**Hawaiian Islands.** Kaua‘i: Hanalei District. Kōke‘e State Park, mile 19 of Highway 550 just below Pu‘u o Kila lookout on southern rim of Kalalau Valey, *D. H. Lorence et al. 6001* (BISH, MO, NY, PTBG, US), Highway 550, mile 19 [southern rim of Kalalau Valley], *T. Flynn et al. 3256* (PTBG).

#### 2. Perrottetia sandwicensis

Taxon classificationPlantaeStylommatophoraStreptaxidae

A. Gray, U. S. Expl. Exped., Phan. 291. 1854.

Fig. 2D–F

##### Type. Hawaiian Islands: O‘ahu.

On mts. behind Honolulu. 1840. *U. S. Expl. Exped. s.n.* (Lectotype: US-16429!; Isolectotype: GH [GH-00050026!]), designated by St. John, Rhodora 87: 570. 1985.

##### Note.

Gray also cited a Gaudichaud collection from the same locality, as well as “also Hawai‘i; along the margin of forests.” The US sheet does not have a specific locality.

PerrottetiasandwicensisA. Grayvar.tomentosa O. Deg. & Greenwell, Rev. S. Am. Bot. 10(1): 25 1951.

##### Type.

Hawaiian Islands: Maui. Mauka of Nawini, S slope of Haleakalā, in gulch in decadent forest, 24 November 1950, *O. Degener , A. B. Greenwell, W. H. Hatheway, Miller, Silva 21119* (Holotype: NY [NY-00337484!]; Isotype: BISH-501715!).

##### Description.

Polygamodioecious shrub or small slender tree 2–6(–8) m tall; branches when fresh red to green, usually glabrous or glabrate, sometimes strigulose or villous-tomentose with light brown hairs 0.1–0.4 mm long. Leaves spirally arranged, shiny, dark green except veins and petioles usually pink, red, or reddish orange; blade chartaceous, ovate, elliptic, or oblong-elliptic, rarely obovate or broadly elliptic (Maui), 7–19 cm long, 2.5–9.5 cm wide [length:width 1.8–2.7:1], adaxially relatively smooth, glabrous, abaxially somewhat paler and glabrate to moderately villosulous (Maui), especially along veins and on young leaves, the hairs yellowish brown, 0.4–0.8 mm long, the secondary veins 8–11 on each side, usually with hair tufts in secondary and tertiary vein axils, the tertiary and quaternary veins usually not prominulous below, apex long- to short-acuminate or sometimes rounded or obtuse (Maui), base cuneate to obtuse or rounded, margin serrate, the teeth 0.3–1 mm long, tips indurated; petiole 1.5–5 cm long, sparsely to moderately pale brown villosulous-hirsute or glabrate; stipules linear-oblong, 1–1.5 mm long, glabrous or puberulent, deciduous. Inflorescences of both sexes axillary or occasionally infrafoliar, paniculate cymes, pyramidal, with two to three (rarely to four on Kaua‘i) degrees of branching, the peduncle (0.5–) 1–4 cm, the staminate 4–7(–12) cm long, 2–6 cm wide, the pistillate 3–5 cm long, 2–3 cm wide; axes glabrate to densely brown villosulous, the hairs 0.1–0.4 mm long, bracts glabrous, triangular-subulate, those on primary branches 0.4–1.0 mm long, those on secondary branches 0.5–1.0 mm long, pedicels with 1–3 bracts 0.5–0.8 mm long; flowers on glabrous or puberulent, articulate pedicels 1–3.5 mm long. Staminate flowers with sepals and petals 5, calyx greenish orange, sometimes red-margined, sepals deltate, 0.5–0.7 mm long, margins entire, petals similar in color and shape to sepals but thinner, 0.6–0.8 mm long, 1–1.2 mm wide, margins ciliate; stamens with filaments 1.5–1.9 mm long, anthers transverse ellipsoid, 0.5–0.6 mm long, 0.6–0.7 mm wide; pistillode 0.5–0.7 mm. Pistillate flowers with sepals and petals 5, bracteolate, with calyx greenish orange to red, sepals deltate, 0.6–1.5 mm long, 0.5– 0.7 mm wide, petals similar in color and shape but thinner, deltate, 0.6–1.1 mm long, margins ciliate; pistil ovoid, 1.0–1.5 mm long, stigma lobes 2, papillose; staminodes 5, 0.3–0.4 mm long. *Infructescence* 5–10 cm long, 3.5–7 cm wide. *Fruit* a depressed-globose berry (2–)4–6 mm in diameter, reddish-green, ripening bright red, or white and red-tinged, glabrous. *Seeds* (2–)4, triangular-globose, 1.3–1.5 mm long, with thin aril, the testa rugose-reticulate, or sometimes only with transverse wavy lines.

##### Distribution.

Hawaiian Islands, endemic to Kaua‘i, O‘ahu, Moloka‘i, Lana‘i, Maui, and Hawai‘i. In mesic and wet forests, c. 300–1250 (–1830 on Maui) m elevation. Flowering was observed from November through July and fruiting from November through August.

##### Common names.

Olomea, Pua‘a olomea, Waimea (Maui).

##### Discussion.

Some collections from East Maui have loosely villosulous stems and lower leaf surface with curling hairs, including the type of Perrottetiasandwicensisvar.tomentosa, but these otherwise correspond to typical *P.sandwicensis*. Since the degree of pubescence intergrades with typical sparsely puberulent or glabrate forms, this variety was not recognized by Wagner et al. 1990, 1999. Pubescence in *P.wichmaniorum* is usually spreading hirtellous with straight hairs and is especially dense on the inflorescence axes and young stems and leaves.

##### Representative specimens examined.

**Hawaiian Islands**: Kaua‘i. Hanalei District, Kōke‘e State Park. Hwy 550 at mile 19, along north side of road, *T. Flynn et al. 3255* (PTBG); Nā Pali-Kona Forest Reserve, upper Hanakoa Valley north of Pihea peak, *T. Flynn & D. H. Lorence 3244* (PTBG, US); Koloa District, Kahili Ridge, *W. L. Stern et al. 3114* (NY, US); Waimea District: Mohihi Valley, *E. H. Bryan 1451* (BISH, US); Kaunuohua Ridge between Kilohana Lookout at Pu‘u o Kila and Pihea, *W. L. Stern & S. Carlquist 1242* (US); Makaha, north facing slopes below and west of Kokio keokeo, *K. R. Wood & M. Query 15806* (PTBG, US). **O‘ahu.** Kona District, Wa‘ahila Ridge, on Ko‘olau summit ridge past Mount Olympus summit, *J. W. Adams & M. Bond 95* (PTBG); ridge E. Nu‘uanu Valley, *C. N. Forbes 1434* (BISH, US); Ko‘olauloa District, Punalu‘u, Castle trail, *B. C. Stone 1158* (US), Waikane-Schofield trail, Waikane side, *T. G. Yuncker 3197* (US); ‘Ewa District, Kipapa Gulch, S ridge, *E. Y. Hosaka 711* (BISH, US); Wai‘anae District, Mt. Ka‘ala, near road, *O. Degener & I. Degener 28015* (US), below summit ridge of head of Makua Valley, *O. Degener & W. Hatheway 21166* (BISH, US); Pu‘u Kalena, *W. R. Donaghho s.n.* (US). **Lana‘i.** Munro Trail, ca. ½ mile from fog drip station, in bottom of feeder valley to Maunalei Gulch, *T. Flynn & D. Palumbo 255* (PTBG); Pu‘u Kole, 14 Jan 1964, *O. Degener & I. Degener 30159* (BISH, US), Ha‘aleiepa‘akai, Kaohai, *H. St. John & A. J. Eames 18788* (BISH, US). **Moloka‘i.** upper end of Hanalilolilo pipe-line, *O. Degener 8971* (BISH, US); west ridge of Honomuni, *H. St. John 25194* (BISH, US). **Maui.** W. Maui; Wailuku District, Kahakuloa Drainage, *K. R. Wood 3144* (AD, NY, PTBG); near last ditchman‘s house on way to Mt. Eke, *O. Degener & H. Wiebke 2561* (US); Lahina District, Honokahau Drainage Basin, *C. N. Forbes 496.M* (US); ravine back of Lahaina, *A. S. Hitchcock 14882* (US); E. Maui, Makawao District, Ahupua‘a of Kalialinui, upper Ko‘olau Gap, 1.5 mi east of Hosmer‘s Grove, *P. K. Higashino 780* (BISH, US); Haleakala, Ko‘olau Gap, valley on west side, 2 mi N of Holua Cave, *H. St. John & A. L. Mitchell 21281* (BISH, US), Hana District, Kaeanae Gap, crater of Haleakalā, *C. N. Forbes 1071.M* (US). **Hawai‘i**. N. Hilo District, Laupāhoehoe Natural Area Reserve, follow Kīlau Stream, *K. R. Wood et al. 3184* (MO, NY, PTBG, WU); S. Hilo District, ‘Ōla‘a State Forest Preserve, 14 Aug 1975, *S. P. Darwin 1218* (PTBG, US); N side of Stainback Hwy. 10 mi. above junction with Hwy. 11, 31 Jan 1968, *D. Herbst 871* (BISH, US); Ka‘ū District, Route 148a, NE of Kilauea, 11 Jul 1961, *O. Degener & I. Degener 28197* (BISH, US).

## Supplementary Material

XML Treatment for Perrottetia wichmaniorum

XML Treatment for Perrottetia sandwicensis

## References

[B1] Ding H (1962) Celastraceae – 1. In: Steenis CGGJ (Ed.) Flora malesiana.Ser. 1, vol. 6. Wolters-Noordhoff, Groningen, 227–291.

[B2] Gray A (1854) United States Exploring Expedition. During the years 1838, 1839, 1840, 1841, 1842. Under the command of Charles Wilkes, U.S.N. vol. XV. Botany. Phanerogamia. Part I. C. Sherman, Philadelphia, 777 pp.

[B3] IUCN (2012) IUCN Red List Categories and Criteria Version 3.1, Second Ed. Prepared by the IUCN Criteria Review Working Group. IUCN, Cambridge. http://www.iucnredlist.org/static/categories_criteria_3_1 [accessed 14.12.2018]

[B4] Lundell CL (1985) Neotropical species of the genus *Perrottetia* (Celastraceae). Phytologia 57(3): 231–238.

[B5] Mabberley DJ (2017) Mabberley‘s Plant-Book. Cambridge University Press, Cambridge, 1102 pp 10.1017/9781316335581

[B6] Matthews ML Endress PK (2005) Comparative floral structure and systematics in Celastrales (Celastraceae, Parnassiaceae, Lepidobotryaceae). Botanical Journal of the Linnean Society 149: 129–194.

[B7] Sánchez Montano R Fernández Alonso JL (2000) Novedades en el género *Perrottetia* (Celastraceas). Anales del Jardín Botánico de Madrid 58(1): 109–116.

[B8] Stevens PF (2001–) Angiosperm Phylogeny Website. Version 14, July 2017 (and more or less continuously updated since). http://www.mobot.org/MOBOT/research/APweb/ [accessed 14.12.2018]

[B9] Wagner WL Herbst DR Sohmer SH (1990) Manual of the flowering plants of Hawai‘i. University of Hawai‘i Press. [Bishop Museum Special Publication 83]

[B10] Wagner WL Herbst DR Sohmer SH (1999) Manual of the flowering plants of Hawai‘i, revised edition with supplement by Wagner, W. L. and D. R. Herbst. University of Hawai‘i Press, 1855–1918. [Bishop Museum Special Publication 97]

[B11] Worberg A Alford MH Quandt D Borsch T (2009) Huerteales sister to Brassicales plus Malvales, and newly circumscribed to include *Dipentodon, Gerrardina, Huertea, Perrottetia*, and *Tapiscia*. Taxon 58: 468–478.

[B12] Zhang L-B Simmons MP (2006) Phylogeny and delimitation of the Celastrales inferred from nuclear and plastid genes. Systematic Botany 31: 122–137.

